# Specialized Care without the Subspecialist: A Value Opportunity for Secondary Care

**DOI:** 10.3390/children5060069

**Published:** 2018-06-04

**Authors:** Eyal Cohen, C. Jason Wang, Barry Zuckerman

**Affiliations:** 1 Department of Pediatrics and Health Policy, Management and Evaluation, University of Toronto, Toronto, ON M5G 1X8, Canada; 2 Division of Pediatric Medicine, The Hospital for Sick Children, Toronto, ON M5G 1X8, Canada; 3 Center for Policy, Outcomes and Prevention and Division of General Pediatrics, Stanford University, Stanford, CA 94305, USA; cjwang1@stanford.edu; 4 Department of Pediatrics, Boston University School of Medicine, Boston, MA 02118, USA; barry.zuckerman@bmc.org

**Keywords:** primary care, subspecialty care, multidisciplinary care, chronic conditions, healthcare delivery, health services research

## Abstract

An underutilized value strategy that may reduce unnecessary subspecialty involvement in pediatric healthcare targets the high-quality care of children with common chronic conditions such as obesity, asthma, or attention deficit hyperactivity disorder within primary care settings. In this commentary, we propose that “secondary care”, defined as specialized visits delivered by primary care providers, a general pediatrician, or other primary care providers, can obtain the knowledge, skill and, over time, the experience to manage one or more of these common chronic conditions by creating clinical time and space to provide condition-focused care. This care model promotes familiarity, comfort, proximity to home, and leverages the provider’s expertise and connections with community-based resources. Evidence is provided to prove that, with multi-disciplinary and subspecialist support, this model of care can improve the quality, decrease the costs, and improve the provider’s satisfaction with care.

## 1. The Rationale for Secondary Care

An important goal in contemporary healthcare is to increase value by improving quality and decreasing cost. Common strategies include reduction of unnecessary hospitalization, tests, and procedures. We suggest a strategy that reduces unnecessary subspecialty involvement as an underutilized value strategy by revisiting a concept that has not been discussed in great detail in healthcare reform: secondary care.

With the growth of more sophisticated integrated healthcare delivery systems, we believe that high-quality care of children with common chronic conditions such as asthma, obesity, chronic constipation, headaches, and attention deficit hyperactivity disorder (ADHD) should take place entirely within a community-based practice. In this article, we outline the advantages to situating such care completely in the community and propose an enhanced role of a general pediatrician supported by the same type of multidisciplinary care and time allotted found in hospital-based clinics to manage a specific chronic condition. Here, we are defining secondary care as specialized visits delivered by primary care providers. In healthcare systems where general practitioners provide primary care (e.g., the United Kingdom or Australia), the term is commonly used to denote consulting general pediatrics [1], but the same concept can also be applied to pediatricians within a primary care setting [2], a model of care that is common in the United States, parts of Europe, and other countries internationally [3]. In the model we are proposing, the interested general pediatrician (or other well-trained pediatric primary care provider) obtains the knowledge, skill and, over time, the experience to manage one or more of these common chronic conditions by creating clinical time and space to provide condition-focused care.

To do this effectively in a community clinic, three key elements are essential: (a) a provider competent with and interest in the underlying condition; (b) a set-up for prolonged chronic-care focused visits during a pre-determined clinic session; and; (c) access, when needed, to an effective multi-disciplinary team. Competence with the underlying condition will require pediatricians to keep up with the literature and obtain ongoing continuing medical education and to experience practice at the top of their skill-set; rare conditions not encountered repeatedly during past training would not be appropriate. In a group practice, different physicians within the group can develop a specific “area of focus” to ensure optimal expertise and economies of scale. Prolonged visits are essential to allow for differentiation from high-throughput primary care practice tempos. A specific slot of time (e.g., one half-day a week) can be carved out within a group practice for a focused clinic (e.g., an obesity clinic). Co-attendance by relevant allied health providers (e.g., a dietician for obesity, a nurse educator for asthma) is essential to ensure comprehensive care for many conditions, although not necessarily all conditions (e.g., ADHD). The cohorting of patients on specific days will help facilitate their attendance and will also help streamline practice workflow.

## 2. How Will This Model Lead to Improved Care?

Locating care in the community promotes familiarity, comfort, proximity to home and, likely, a good fit culturally for the patient and parents. Primary care clinicians providing high-quality chronic care for common conditions will help free up limited subspecialty capacity and bottlenecks to care for sicker and/or more complex patients; this is especially important in medically underserved areas. For instance, patients with functional gastrointestinal disorders predominate in some gastroenterology clinics [4], which may be reflective of a lack of availability of a generalist to manage these patients effectively in secondary care.

Focusing disease management in a primary care setting also leverages the provider’s and allied health professionals’ expertise and connections with community-based resources. Knowledge of local school-, community center-, or other community-based supports promotes the optimal management of any behavioral condition, provides supports for the enactment of nutritional and/or exercise plans, and also provides an outlet for meaningful patient advocacy. Alternative models of primary care/subspecialty integration, such as those that embed generalists in subspecialty clinics [5], may lack this critical community-based connectivity.

A community-based provider also has the ideal foundation for the establishment of longitudinal trusting connections with a patient and their family. Successful chronic disease management relies on productive relations between engaged patients/families with prepared and proactive clinicians. Who is better positioned to build these relationships than a community based primary care provider who has the expertise, time, and team?

We believe there is an opportunity for decreased cost with a growth of secondary care. Most subspecialists practice in hospital-based settings, which leads to substantially increased expenses due to facility fees. Further, for some conditions, subspecialists may be more likely to order potentially unnecessary diagnostic testing due to the ready availability of such testing modalities, the lack of knowledge of the patient’s circumstances, and the follow-up capacity and/or institutional financial incentives to do more tests [6].

Some may argue that disease management by a generalist with less expertise and training may be lower quality care. While a potential concern, this is not supported empirically. Outcomes of children with Type 1 diabetes in Australia and Canada who are usually managed by specialists in other countries like the United States seem to be similar whether an endocrinologist or a general pediatrician leads their care [7,8]. Most generalists without access to subspecialty services also report a high comfort level managing some patients who are usually cared for by specialists [9]. With ongoing experience with the specific medical condition, the skill and comfort level of generalists are likely to increase.

Effective connectivity to specialized care is still essential for this proposed model to succeed. While the vast majority of children with obesity or asthma or ADHD can be cared for in the community, a small, but important subset will undoubtedly benefit from specialty care (e.g., obese child with medical comorbidities, children with asthma with repeated life-threatening exacerbations, children with ADHD and multiple behavioral comorbidities). To be effective, community practices that provide specialized care will have to be well linked within a regionalized system of care. This is essential not just for referrals but to also provide educational support to the general pediatrician. 

Fortunately, there are templates to help facilitate such support. Project ECHO (extension for community healthcare outcomes) is a rapidly growing hub-and-spoke knowledge sharing network, whereby expert teams use telemedicine interfaces to conduct virtual clinics and deliver educational curricula to community providers [10]. Originally conceived for the community-based management of hepatitis C in New Mexico, models such as Project ECHO provide the added benefit of disseminating specialized knowledge for primary care providers and empowering patients and community-based providers to partner in delivering high-quality community care. These networks have already been established for primary care providers caring for a number of pediatric chronic conditions like Sickle Cell Disease [11]. Other initiatives focused on promoting high value care like Choosing Wisely can also be leveraged to spread knowledge and uptake of secondary care [12].

What barriers may prevent the development of these models? Financial incentives are lacking, particularly in fee-for-service environments that incentivize high volume, manifesting as well-child care in primary care and/or low complexity subspecialist care. In the changing landscape driven by healthcare reform, this can be addressed in models like accountable care organizations that are growing in popularity, given the significant amount of money that payers can save, particularly in integrated delivery systems. We also believe most primary care clinicians with good training will love to have the opportunity and satisfaction of developing special competency and the professional satisfaction that accompanies it. This will take some investment: from primary care clinicians in terms of their own training via coursework and conferences, from organizers and accreditors by continuing medical education activities, and from subspecialists who have an essential role in building up these competencies. It will also take a partnership with patients and families who may perceive that they are being denied subspecialist access and may not appreciate the potential benefits of this model in terms of convenience, shorter wait times, and other factors. We hope the health system can recognize and mobilize to implement secondary care. With the effective implementation of delivery systems, the stars, in terms of quality, cost, and pediatrician satisfaction, are all aligned.
